# How May Coexisting Frailty Influence Adherence to Treatment in Elderly Hypertensive Patients?

**DOI:** 10.1155/2019/5245184

**Published:** 2019-01-06

**Authors:** Bartosz Uchmanowicz, Anna Chudiak, Izabella Uchmanowicz, Grzegorz Mazur

**Affiliations:** ^1^Department of Clinical Nursing, Faculty of Health Sciences, Wroclaw Medical University, 5 Bartla Street, 51-618 Wroclaw, Poland; ^2^Department and Clinic of Internal and Occupational Diseases and Hypertension, Jan Mikulicz-Radecki University Teaching Hospital in Wroclaw, 213 Borowska Street, 50-556 Wrocław, Poland

## Abstract

**Background:**

Hypertension is considered to be the most common condition in the general population. It is the most important risk factor for premature deaths in the world. Treatment compliance at every stage is a condition for successful antihypertensive therapy, and improving the effectiveness of treatment is a major goal in preventing cardiovascular incidents. Treatment noncompliance and lack of cooperation stem from numerous problems of older age, including frailty syndrome.

**Objective:**

To evaluate the effect of frailty syndrome on treatment compliance in older patients with hypertension.

**Methods:**

The study sample consisted of 160 patients (91 women, 69 men) with hypertension aged 65 to 78 (mean = 72.09, SD = 7.98 years), hospitalized at the University Clinical Hospital due to exacerbation of disease symptoms. Standardised research tools were used: the Tilburg Frailty Indicator questionnaire and the questionnaire for the assessment of treatment compliance in patients with hypertension, the Hill-Bone Compliance to High Blood Pressure Therapy Scale.

**Results:**

Frailty syndrome was diagnosed in 65.62% of patients: 35.62% with mild, 29.38% with moderate, and 0.62% with severe frailty. The treatment compliance was 36.14%. The prevalence of the FS and its three components (physical, psychological, social) significantly affected (p <0.05) the global score of the Hill-Bone Compliance to High Blood Pressure Therapy Scale and all subscales: “reduced sodium intake”, “appointment keeping”, and “antihypertensive medication taking”.

**Conclusions:**

The coexistence of frailty syndrome has a negative impact on the compliance of older patients with hypertension. Diagnosis of frailty and of the associated difficulties in adhering to treatment may allow for targeting the older patients with a poorer prognosis and at risk of complications from untreated or undertreated hypertension and for planning interventions to improve hypertension control.

## 1. Introduction

Hypertension (HT) is the most important risk factor for premature deaths worldwide, and blood pressure levels (BP) indicate a linear correlation between mortality and morbidity in cardiovascular diseases (heart attack, stroke, heart failure, and peripheral artery disease) and renal failure [1]. HT is considered to be the most common disease in the general population, affecting more than 20% of the population of highly developed countries. The National Health and Nutrition Examination Survey (NHANES) data estimate that, in the United States, HT prevails in 33.5% of the adult population [2]. In the United Kingdom, the proportion of patients is higher and amounts to 42% [3]. Epidemiological data highlight the core of the problem, as the number of patients with HT in Poland is 2% higher than 10 years ago. Currently, HT prevails in about 32% of the Polish population. The incidence of HT increases with age, and the available data indicate that 1 million people > 80 years of age suffer from HT [1]. It has also been shown that the proportion of patients with HT among the retired population is higher than in the general population and is estimated at 58% [4].

A successful antihypertensive treatment depends on the treatment compliance (“compliance” and “adherence”), and improving the effectiveness of treatment of hypertension is a major goal in the prevention of cardiovascular incidents. Unfortunately, only 30% of patients with HT comply with treatment plans; in Poland, the fraction is estimated at 5-15% [5, 6]. The terms “compliance” and “adherence” refer to the factors determining the successful treatment effects in terms of the patient-doctor relationship. It means “the extent to which patients comply with medical plans, diet, lifestyle modifications” [7]. Non-compliance with the treatment plan is one of the most important causes underlying insufficient BP control. Other reasons of non-compliance with treatment plans and lack of cooperation include numerous problems of older age, such as frailty syndrome (FS).

Correlation of various concomitant diseases, older age, and disability may result in the development of FS. A widely used definition of FS proposed by Fried et al. [8] states it is “a biologic syndrome of decreased reserve and resistance to stressors, resulting from cumulative declines across multiple physiologic systems and causing vulnerability to adverse outcomes”. The American Geriatrics Society (AGS) identified major risk factors for FS: reduced resistance to stressors and reduction of adaptive and physiological reserves of many organs in the body, including endocrine disorders and dysfunctions of the immune system [9]. In Europe, the prevalence of FS is estimated at the level of 17-20% of the population. The number of people with FS is increasing with age, reaching nearly 40% in the group > 85 years of age. The studies to date revealed the 4-times higher mortality rate in people with FS in the annual observation [10].

The older population comprised a special group of hypertensive patients due to the occurrence of ageing-related processes. An isolated hypertension is observed in systolic blood pressure (SBP), when diastolic blood pressure (DBP) values are below 90 mmHg. In the older population, SBP is a more significant risk factor for cardiovascular events than DBP. Chronically higher values of SBP lead to left ventricular hypertrophy [11]. Despite this knowledge, it should be emphasised that antihypertensive treatment in older patients is still debated. The most recent European guidelines in this group of patients recommend antihypertensive treatment when values of SBP reach ≥160 mmHg, with a systolic target between 140 and 150 mmHg [12]. Also, a significant difference was shown in the use of antihypertensive drugs, except for the use of angiotensin‐converting enzyme inhibitors (ACEIs) and calcium channel blockers (CCBs). Older patients with FS use more diuretics followed by *β*‐blockers [13].

Frailty is defined as a biologic syndrome of decreased reserve and resistance to stressors, resulting from cumulative declines across multiple physiologic systems, causing vulnerability to adverse outcomes [14]. Recent studies have shown that FS is a common geriatric syndrome characterized by increased risk of disability, hospitalization, and mortality [15–18]. So far, the association between HT and FS in older adults remains unclear. Aprahamian et al. [13] in their cross-sectional study among 619 older adults showed that HT is more prevalent in frail older patients and was significantly associated with FS. Kang et al. [19] analysed data of 4,352 older adults and they indicated that HT prevalence was higher in frail elderly (67.8%) than pre-frail (60.8%) or robust elderly (49.2%). Warwick et al. [20] raise some remarks indicating the lack of evidence of an interaction between the effect of treatment for HT and FS as measured by the frailty index (FI). However, it should be emphasised that the management of HT in frail older people is a newly emerging problem [21] and in this context should be undoubtedly considered as a warning sign motivating a preventive actions [22].

Considering the physiological alterations resulting from ageing and the presence of multiple comorbidities, treatment of hypertension in older patients poses a significant challenge to treatment teams [21]. In clinical practice, special attention should be paid to frail older patients, who should receive tailored treatment. Moreover, effective antihypertensive therapy in those patients younger than 80 years should not be discontinued simply because that age milestone has been reached [23]. It should be noted that multidisciplinary approach and treatment individualization can be beneficial in the treatment of hypertension in the older patients with FS [24].

The aim of the study was to evaluate the influence of FS on treatment compliance in older patients with hypertension. It should be pointed out that our intention was to focus more on psychological and social factors than physical capacity including muscle strength, gait speed, postural control, and functional parameters.

## 2. Materials and Methods

### 2.1. Study Design and Participants

This was a cross-sectional study involving 160 patients being hospitalized in the Clinic of Angiology, Hypertension, and Diabetology at the University Clinical Hospital in Wrocław, Poland, due to exacerbation of disease symptoms. Participation was anonymous and voluntary and patients were told that they could withdraw at any time.

### 2.2. Selection Criteria

The inclusion criteria for the study were as follows: hypertension lasting at least 1 year; age ≥ 65 years; hypertension treated with at least 1 antihypertensive drug; the patient's consent to participate in the study. Hypertension diagnosis was performed according to the Seventh Joint National Committee (JNC-7) classification of blood pressure for adults, as follows: normal for <120 SBP and <80 DBP, prehypertension for 120-139 SBP and 80-89 DBP, hypertension stage 1 for 140-159 SBP or 90-99 DBP, and hypertension stage 2 for ≥160 SBP or ≥100 DBP [25].

### 2.3. Ethical Considerations

The study was approved by the Bioethics Committee of Wroclaw Medical University (no. KB–114/2016). Signed informed consent forms were obtained from all patients who were included in the study. The study was conducted in accordance with the Helsinki Declaration and the European Medicines Agency Guidelines for Good Clinical Practice. The dignity and rights of participants were respected at all times.

### 2.4. Research Tools

Standardised research tools were used in the study: the Tilburg Frailty Indicator (TFI) questionnaire and the questionnaire for antihypertensive treatment compliance, the Hill-Bone Compliance to High Blood Pressure Therapy Scale (HBCHTS).

The Tilburg Frailty Indicator (TFI) questionnaire by Gobbens et al. [26] consists of 15 questions in two parts. Part A relates to health determinants of FS such as sociodemographic characteristics (sex, age, marital status, country of origin, educational level, and monthly income), lifestyle, multimorbidity, life events, and living environment. Part B contains the main components of FS including 8 physical (weight, daily life, walking, hearing, vision, strength, and tiredness), 4 psychological (memory, mood, anxious, and coping with problems), and 3 social components (living alone, people around, and support). The maximum score is 15 points; however, the diagnosis of FS is confirmed by obtaining at least 5 points. The following cut-off scores were used to classify the severity of frailty syndrome: not frail (0–4); mildly frail (5–8); moderately frail (9–12); severely frailty (13–15) [26]. This study used the Polish version of the questionnaire [27].

The Hill-Bone Compliance to High Blood Pressure Therapy Scale (HBCHTS) is comprised of 14 questions in 3 subscales: “reduced sodium intake” (3 questions), “appointment keeping” (2 questions), and “medication taking” (9 questions). The respondent has the following answers: “none of the time”, “some of the time”, “most of the time”, “all the time”, “not applicable”, and “do not know”. The maximum score is 56 points, meaning the higher the score, the poorer the treatment compliance. The Polish version of the questionnaire was used in this study [28].

### 2.5. Statistical Methods

Statistical analysis of quantitative variables was performed by calculating means, standard deviations, medians, quartiles, and minimum and maximum values. Analysis of qualitative variables was performed by calculating the number of times and percentage of occurrences of each value. The correlation between two quantitative variables was analyzed using the Spearman's coefficient. Correlation coefficients were interpreted as follows: |r| ≥ 0.9, very strong correlation; 0.7 ≤ |r| < 0.9, strong correlation; 0.5 ≤ |r| < 0.7, moderately strong correlation; 0.3 ≤ |r| < 0.5, weak correlation; |r| ≥ 0.3, very weak (negligible) correlation. Normal distribution of variables was verified using the Shapiro-Wilk test. To further illuminate the relationship between frailty syndrome and patient's compliance, linear regression was conducted. Covariates were selected with a backward stepwise procedure, starting with full model (with all possible covariates) and removing the least significant one (excluding frail variable) until only statistically significant variables (and frail variable) were left. The significance level was set at 0.05. Therefore, all p values below 0.05 were interpreted as indicating significant correlation. The analysis was carried out with R 3.3 software.

## 3. Results

### 3.1. Sample Characteristics

The study of the influence of FS on treatment compliance was carried out among 160 patients (91 women, 69 men) with HT aged from 65 to 78 years (mean = 72.09, SD = 7.98 years). Basic sociodemographic and clinical characteristics are presented in Table 1.

### 3.2. Analysis of the Prevalence of Frailty Syndrome with TFI

FS was diagnosed in 105 out of 160 patients (65.62%). TFI has 3 subscales. Relatively, the most important contributor of frailty in the group studied was the social component (mean 1.86 out of possible 3 points, which translates into 61.88% of the maximum score). The physical component was slightly less important (mean 3.84 points out of 8 points, 48.05% of the maximum score), and the psychological component was the least important (on average 1.24 points out of 4, 31.09% of the maximum score). Detailed data are presented in Table 2.

The analysis of the TFI score allowed the researchers to grade FS severity into mild, moderate, and severe. Thus, 57 out of 160 survey participants (35.62%) had mild FS, 47 participants (29.38%) had moderate FS, and 1 participant (0.62%) had severe FS, determined with the TFI. Detailed data are presented in Table 3.

### 3.3. Assessment of Treatment Compliance with HBCHTS

The global score of this questionnaire ranged between 14 and 56 points. The higher the score, the poorer the treatment compliance. The mean score obtained by study participants was 20.24 (SD = 4.01) and ranged from 14 to 32 points. The median was 19.5 points, meaning half of the participants obtained less, and half obtained more than 19.5 points. The first and third quartiles were 17 and 22, respectively, so the mean score in the studied group ranged between 17 and 22 points. Every Hill-Bone subscale has a different number of questions. Hence, each scale has a different range of values.

The “sodium intake” subscale contains three questions (range 3-12), and the obtained mean score was 4.79 (SD = 1.08). The “appointment keeping” subscale contains 2 questions (range 2-8). On this subscale, the mean score was 3.35 (SD = 1.18). The “medication taking” subscale contains 9 questions (range 9-36) and the mean score obtained was 12.1 (SD = 3.05). Detailed data are presented in Table 4.

### 3.4. Influence of FS on Treatment Compliance in HBCHTS

The prevalence of FS and its three components (physical, psychological, and social) were significantly correlated with (p <0.05) the global score of the HBCHTS questionnaire and all subscales: “reduced sodium intake”, “appointment keeping”, and “antihypertensive medication taking”. All these correlations were positive; i.e., the higher the score on the TFI questionnaire, the higher the score on the HBCHTS questionnaire.

The correlation between FS (global score of TFI), physical components, and treatment compliance was moderate and, in the case of psychological and social components, weak. Detailed data are presented in Table 5.

The backward stepwise method has selected three variables that significantly affect the relationship between frailty and compliance. They are sex, age, and education. After considering these variables in the model, the impact of frailty becomes irrelevant. Compliance of patients with mild and severe frail does not differ significantly from those with no frail. The linear regression model showed that the independent predictors of the global score of the HBCHTS questionnaire are as follows (p <0.05): (1) sex: in comparison with female, male increases the global score of the HBCHTS questionnaire (decreases compliance) by an average of 1.23 points; (2) education: compared with none or basic, a secondary education decreases the global score of the HBCHTS questionnaire (increases compliance) by an average of 0.95 points, and a higher one decreases the global score of the HBCHTS questionnaire (increases compliance) by an average of 2.02 points; (3) age: each additional year of life lowers the global score of the HBCHTS questionnaire (decreases compliance) by an average of 0.20 points. Detailed data are presented in Table 6.

## 4. Discussion

The coexistence of the FS may have a detrimental effect on the course of the disease and, additionally, the level of treatment compliance [29, 30].

In our study, FS affected 65.62% of the group studied. FS influenced the level of treatment compliance, cooperation during the study, and the global score on the Hill-Bone questionnaire. It also adversely affected the questionnaire subscales: “reduced sodium intake”, “appointment keeping”, and “antihypertensive medication taking”. There are no studies on the effect of FS on perseverance in the treatment process. However, the Medicaid study emphasized that only 20% of hypertensive patients over 65 comply with the treatment regimen, which might result from the aging processes of the body, including FS [31].

Concurrent frailty and cognitive impairment in older patients are associated with a worse perception of health, increased number of comorbidities, and social isolation of the patient [14]. The coincidence of the FS can, therefore, affect the inferior adherence to the therapeutic recommendations as confirmed in our own study [32].

There are a few studies which measure the association between the components of the geriatric syndrome and adherence to treatment. Papers that discuss associations between frailty and adherence are based on populations with diseases rather than hypertension [33, 34]. There is a discussion in the available literature regarding the impact of FS on adherence [33]. In a study by Koizumi et al. [35], frailty in hypertensive patients was associated with limited physical activity, lower body weight, difficulties in ingesting solid foods and performing daily activities, and limitations in performing complex activities of daily living, correlated with the prevalence, treatment and control of hypertension. Also, our previous study showed that FS exerts a significant effect on adherence to treatment recommendations among older patients suffering from HT. We proved that a treatment compliance is negatively affected in male patients, in those with lower education, as well as patients with lower TFI scores [36].

Other significant issues in older patients include polypharmacy, increased risk of falls, and cognitive impairment. Antihypertensive treatment of older patients in line with the European Society of Hypertension (ESH) and European Society of Cardiology (ESC) guidelines [37] largely reduces the risk of stroke and mortality from cardiovascular incidents. Without a doubt, treatment of hypertension in older patients poses a significant challenge to treatment teams (polypharmacy, affected pharmacokinetics, atherosclerosis, reduced kidney performance, osteoporosis, and cognitive impairment). Moreover, older hypertensive patients are a special risk group for orthostatic hypertension, polypharmacy, falls, and cognitive impairment [21].

Identifying the causes of nonadherence to pharmaceutical treatments is a very important factor in planning therapeutic interventions aimed at increasing control, preventing complications, and improving long-term outcomes and any adverse effects of treatment. Precise identification of contributors to low medication adherence is crucial for improving treatment effectiveness and for distinguishing those patients in need of additional supervision in order to decrease the risk of complications from untreated hypertension [21].

## 5. Study Limitations

There is a one key study limitation that should be discussed. Namely, only a self-reported test identifying FS (TFI tool) was used in the present study. It should be pointed out that some functional tests such as hand grip strength testing with a dynamometer, walk speed assessment using Timed Up and Go test or Gait Speed test, postural control using tensometric platform, and muscle strength testing with an isokinetic device or surface electromyography, should be considered in the future studies as valuable tools for better characterization of frail patients. However, our intention was to focus more on psychological and social factors than physical capacity. The last potential limitation is the fact that the study sample consisted of elderly hypertensive patients from a single outpatient centre. Thus, our findings should be carefully extrapolated to the multicentre or institutional studies.

## 6. Conclusion

The coexistence of FS has a negative impact on the compliance of older patients with hypertension. Diagnosis of frailty and of the associated difficulties in adhering to treatment may allow for targeting the older patients with a poorer prognosis and at risk of complications from untreated or undertreated hypertension and for planning interventions to improve hypertension control.

## Figures and Tables

**Table 1:** Characteristics of the study group (sociodemographic and clinical variables).

**Characteristics**		**Mean (SD)**	**Median (Q1 – Q3)**
Age [years]		72.09 (7.98)	69 (65 – 78)

Systolic blood pressure [mmHg]		141.57 (16.95)	140 (130 – 150)

Diastolic blood pressure [mmHg]		84.89 (12.86)	90 (80 – 90)

Duration of disease [years]		14.31 (9.19)	14 (7 – 17.25)

**Characteristics**		**N**	%

Sex	Women	91	56.88%
	Men	69	43.12%

Marital status	Married / Living together	84	52.50%
	Unmarried	7	4.38%
	Separated / Divorced	5	3.12%
	Widowed	64	40.00%

Education	None or basic	55	34.38%
	Secondary	60	37.50%
	Higher	45	28.12%

Occupational activity	Employed	37	23.12%
	Retired	106	66.25%
	Annuitant	17	10.62%
	Unemployed	0	0.00%

BMI	Underweight (17 – 18.5)	1	0.62%
	Normal weight (18.5 – 25)	42	26.25%
	Overweight (25 – 30)	71	44.38%
	Obese class I (30 – 35)	31	19.38%
	Obese class II (35 – 40)	10	6.25%
	Obese class III ( > 40)	5	3.12%

JNC-7 classification of HT	Prehypertension	39	24.38%
	Stage I HT	90	56.25%
	Stage II HT	31	19.38%

Co-morbidities*∗*	Diabetes	86	53.75%
	Hypercholesterolemia	49	30.63%
	Coronary disease	47	29.38%
	Renal failure	20	12.50%
	Rheumatic diseases	27	16.88%

Medications administration*∗*	Alpha-adrenolytic	6	3.75%
	Beta-adrenolytic	45	28.12%
	Calcium channel blocker (CCB)	46	28.75%
	Angiotensin receptor blocker (ARB)	14	8.75%
	Beta-blocker (BB)	4	2.50%
	Loop diuretic (LD)	3	1.88%
	Thiazide diuretic (TD)	62	38.75%
	Angiotensin-converting-enzyme inhibitors (ACEI)	105	65.62%

Therapy	Monotherapy	72	45.00%
	Polytherapy	88	55.00%

*∗*Multiple choice questions, percentages don't add up to 100%.

ACEI: angiotensin-converting-enzyme inhibitors; ARB: angiotensin receptor blocker; BB: beta-blocker; BMI: body mass index; CCB: calcium channel blocker; HT: hypertension; JNC: Joint National Committee; LD: loop diuretic; N: number of patients; Q1-Q3: quartile 1^st^-3^rd^; SD: standard deviation; TD: thiazide diuretic.

**Table 2:** Analysis of the occurrence of frailty syndrome using the TFI questionnaire.

**TFI**			**N**				%	
Lack of FS			55				34.38%	
Presence of FS			105				65.62%	

**TFI subscales**	N	**M**	**SD**	**Me**	**Min**	**Max**	**Q1**	**Q3**

Physical components	160	3.84	1.88	4	0	7	2	5
Psychological components	160	1.24	0.87	1	0	4	1	2
Social components	160	1.86	0.78	2	0	3	1	2

M: mean; Max: maximum; Me: median; Min: minimum; N: number of patients; Q1-Q3: quartiles 1^st^-3^rd^; SD: standard deviation; TFI: Tilburg Frailty Indicator.

**Table 3:** The assessment of frailty syndrome.

**TFI**	**N**	%
Mild FS	57	35.62%
Moderate FS	47	29.38%
Severe FS	1	0.62%

FS: frailty syndrome; N: number of patients; TFI: Tilburg Frailty Indicator.

**Table 4:** Evaluation of compliance with therapeutic recommendations using a HBCHTS questionnaire.

**HBCHTS –** **Global score**	**N**	**M**	**SD**	**Me**	**Min**	**Max**	**Q1**	**Q3**
	160	20.24	4.01	19.5	14	32	17	22
**Subscale**	**N**	**M**	**SD**	**Me**	**Min**	**Max**	**Q1**	**Q3**

Reduced sodium intake	160	4.79	1.08	5	3	8	4	5
Appointment keeping	160	3.35	1.18	3	2	6	2	4
Antihypertensive medication taking	160	12.1	3.05	11	9	24	10	14

HBCHTS: Hill-Bone Compliance to High Blood Pressure Therapy Scale; M: mean; Max: maximum; Me: median; Min: minimum; N: number of patients; Q1-Q3: quartiles 1^st^-3^rd^; SD: standard deviation.

**Table 5:** The effect of the frailty syndrome on compliance with therapeutic recommendations.

**HBCHTS**	**TFI**	**Correlation**			
		**Correlation Coefficient**	**p**	**Direction**	**Strength**
Global score	Global score	0.429	<0.001	positive	moderate
	Physical components	0.376	<0.001	positive	moderate
	Psychological components	0.213	0.007	positive	weak
	Social components	0.255	0.001	positive	weak

Reduced sodium intake	Global score	0.429	<0.001	positive	moderate
	Physical components	0.376	<0.001	positive	moderate
	Psychological components	0.213	0.007	positive	weak
	Social components	0.255	0.001	positive	weak

Appointment keeping	Global score	0.429	<0.001	positive	moderate
	Physical components	0.376	<0.001	positive	moderate
	Psychological components	0.213	0.007	positive	weak
	Social components	0.255	0.001	positive	weak

Antihypertensive medication taking	Global score	0.429	<0.001	positive	moderate
	Physical components	0.376	<0.001	positive	moderate
	Psychological components	0.213	0.007	positive	weak
	Social components	0.255	0.001	positive	weak

HBCHTS: Hill-Bone Compliance to High Blood Pressure Therapy Scale; p: *p* value; TFI: Tilburg Frailty Indicator.

**Table 6:** The effect of frailty syndrome on compliance with therapeutic recommendations including gender, age and educational status of the patients.

**Variable**		**R**	**95**%** CI**		**p**
TFI	Not frail – ref.				
	Mildly frail	1.086	-0.287	2.459	p=0.12
	Moderately or severe frail	0.68	-0.97	2.33	p=0.417

Gender	Women – ref.				
	Men	1.232	0.168	2.296	p=0.024

Age [years]		0.203	0.119	0.288	p<0.001

Education	None or basic – ref.				
	Secondary	-0.953	-2.249	0.343	p=0.148
	Higher	-2.022	-3.491	-0.554	p=0.007

CI: confidence interval; p: *p* value; R: regression parameter; TFI: Tilburg Frailty Indicator.

## Data Availability

All data underlying the findings described in this article is fully available to all interested researchers upon request.

## Abbreviations

ACEIs: Angiotensin‐converting enzyme inhibitors
AGS: The American Geriatrics Society
CCBs: Calcium channel blockers
DBP: Diastolic blood pressure
ESC: The European Society of Cardiology
ESH: The European Society of Hypertension
FI: Frailty index
FS: Frailty syndrome
HBCHTS: The Hill-Bone Compliance to High Blood Pressure Therapy Scale
HT: Hypertension
JNC-7: Seventh Joint National Committee
NHANES: The National Health and Nutrition Examination Survey
SBP: Systolic blood pressure
TFI: The Tilburg Frailty Indicator.
